# Ultrarapid and high-resolution HLA class I typing using transposase-based nanopore sequencing applied in pharmacogenetic testing

**DOI:** 10.3389/fgene.2023.1213457

**Published:** 2023-06-23

**Authors:** Nampeung Anukul, Piroon Jenjaroenpun, Chonticha Sirikul, Natnicha Wankaew, Pattaraporn Nimsamer, Ekkapong Roothumnong, Manop Pithukpakorn, Nipapan Leetrakool, Thidathip Wongsurawat

**Affiliations:** ^1^ Division of Transfusion Science, Department of Medical Technology, Faculty of Associated Medical Sciences, Chiang Mai University, Chiang Mai, Thailand; ^2^ Division of Medical Bioinformatics, Research and Innovation Department, Faculty of Medicine Siriraj Hospital, Mahidol University, Bangkok, Thailand; ^3^ Siriraj Long-read Lab (Si-LoL), Faculty of Medicine Siriraj Hospital, Mahidol University, Bangkok, Thailand; ^4^ Division of Medical Genetics, Department of Medicine, Faculty of Medicine Siriraj Hospital, Mahidol University, Bangkok, Thailand; ^5^ Siriraj Genomics, Faculty of Medicine Siriraj Hospital, Mahidol University, Bangkok, Thailand; ^6^Blood Bank Section, Maharaj Nakorn Chiang Mai Hospital, Faculty of Medicines, Chiang Mai University, Chiang Mai, Thailand; ^7^Department of Biomedical Informatics, College of Medicine, University of Arkansas for Medical Sciences, Little Rock, AR, United States

**Keywords:** human leukocyte antigen, long-read sequencing, nanopore, turnaround time, pharmacogenomics, Thai, HLA-B*15:02, ADR

## Abstract

Nanopore sequencing has been examined as a method for rapid and high-resolution human leukocyte antigen (HLA) typing in recent years. We aimed to apply ultrarapid nanopore-based HLA typing for HLA class I alleles associated with drug hypersensitivity, including HLA-A*31:01, HLA-B*15:0*2*, and HLA-C*08:01. Most studies have used the Oxford Nanopore Ligation Sequencing kit for HLA typing, which requires several enzymatic reactions and remains relatively expensive, even when the samples are multiplexed. Here, we used the Oxford Nanopore Rapid Barcoding kit, which is transposase-based, with library preparation taking less than 1 h of hands-on time and requiring minimal reagents. Twenty DNA samples were genotyped for HLA-A, -B, and -C; 11 samples were from individuals of different ethnicity and nine were from Thai individuals. Two primer sets, a commercial set and a published set, were used to amplify the *HLA-A*, -*B*, and -*C* genes. HLA-typing tools that used different algorithms were applied and compared. We found that without using several third-party reagents, the transposase-based method reduced the hands-on time from approximately 9 h to 4 h, making this a viable approach for obtaining same-day results from 2 to 24 samples. However, an imbalance in the PCR amplification of different haplotypes could affect the accuracy of typing results. This work demonstrates the ability of transposase-based sequencing to report 3-field HLA alleles and its potential for race- and population-independent testing at considerably decreased time and cost.

## 1 Introduction

Human leukocyte antigen (HLA) is an antigen on the surface of white blood cells and almost all types of nucleated cells in humans. HLA is divided into three classes: class I, class II, and class III. However, only HLA class I and class II are involved in antigen presentation in the immune system. Classical HLA class I includes HLA-A, -B, and -C, and HLA class II includes HLA-DR, -DQ, and -DP. HLA is not only a factor in differentiating between self- and non-self-antigens, which is important in organ or stem-cell transplantation and blood transfusion but also a marker for disease association and drug hypersensitivity (Kloypan et al., 2021). To assist physicians in choosing the appropriate organ or stem-cell donor, identifying the cause of some diseases, and screening before drug administration to prevent severe allergic reactions, accurate HLA typing at either the antigen or allele level is highly important. To that end, this study focused primarily on drug hypersensitivity applications.

Drug hypersensitivity is an adverse reaction to a drug. Patients can experience symptoms that range from mild, such as a rash and urticaria, to severe and potentially life threatening, such as Stevens–Johnson syndrome (SJS), toxic epidermal necrolysis (TEN), drug rash with eosinophilia and systemic symptoms (DRESS) or drug hypersensitivity syndrome, and acute generalized exanthematous pustulosis (Pavlos et al., 2012). Many studies have shown that severe cutaneous adverse reactions (SCARs) are related to the genetic characteristics of the human leukocyte antigen gene. The *HLA* alleles most frequently involved in drug hypersensitivity reactions are class I. Based on original experiments together with increasing utilization of genome-wide association study (GWAS) approaches and meta-analysis studies, a number of SNPs were reported and confirmed HLA associations, such as HLA-B*57:01, associated with abacavir treatment in patients with HIV (Mallal et al., 2008; Yun et al., 2012; Sousa-Pinto et al., 2015); HLA-B*15:02, associated with carbamazepine treatment in patients with epilepsy (Chung et al., 2004; Tangamornsuksan et al., 2013); and HLA-B*58:01, associated with allopurinol treatment in patients, primarily in Asian populations, including Han Chinese, Korean, and Thai (Tassaneeyakul et al., 2009; Sukasem et al., 2016), and globally (Keller et al., 2018). From GWAS, HLA-B*58:01 associations have been confirmed in both European and Japanese SJS/TEN patients and suggested to be a common biomarker for the global population (Génin et al., 2011; Tohkin et al., 2011).

In Thailand, HLA screening is included in the National Public Health Policy (Kloypan et al., 2021; Sukasem et al., 2023) and HLA genotyping has been implemented in routine clinical practice to maximize drug safety, initially for prescription drugs (Koomdee et al., 2022). However, the selection of HLA markers should be well-considered, especially in the case of ethnic-specific biomarkers like the HLA-B*15:02 allele. In Thai, Han Chinese, and Indian groups, the HLA-B*15:02 allele is a marker for carbamazepine medication reactions, although no such association has been noted in Japanese or European populations. The HLA-A*31:01 allele, however, was significantly linked to carbamazepine-induced SCARs in populations from Japan, Africa, and Europe instead. This allele was confirmed by one of the first GWAS conducted for carbamazepine-induced adverse drug reactions in the Japanese (Ozeki et al., 2011). The HLA-B75 serotype also needs to be concerned in carbamazepine drug users, where even HLA-B*15:02 was not detected because carbamazepine-induced SCARs have been reported in patients who carry either HLA-B*15:08, HLA-B*15:11, or HLA-B*15:21 alleles (Jaruthamsophon et al., 2017; Kloypan et al., 2021). Moreover, a strong association was found between DRESS and HLA-A*33:03, but not with HLA-B*15:02 (Satapornpong et al., 2020). Therefore, all alleles associated with SCARs should be included in the genetic testing panel. In the case of carbamazepine treatment, ideally, at least six HLA alleles should be screened (HLA-B*15:02, HLA-B*15:08, HLA-B*15:11, HLA-B*15:21, HLA-A*31:01, and HLA-A*33:03). In addition, population migration is rapidly increasing worldwide, affecting the prevalence of *HLA* alleles in certain populations and potentially increasing HLA polymorphism and generating novel alleles. Selecting the HLA screening method is crucial—the method must be race- or population-independent, simple and rapid, and have the potential to detect novel alleles.

Several molecular methods have been widely used in routine HLA laboratory tests, depending on the cost and turnaround time, including polymerase chain reaction with sequence-specific primers (PCR-SSPs) or oligonucleotide probes (PCR-SSOPs), real-time PCR (qPCR), and PCR sequence-based technique (SBT). Here, we tested the long-read sequencing technology with nanopore-based methods because of its advantages over other methods. The MinION nanopore sequencer (Oxford Nanopore Technologies, ONT) is a portable sequencer that is easy to transport, generates long-read sequences in real-time, decreases turnaround time, does not require sample pooling, does not require a third-party library preparation kit, and has undergone continual development to improve accuracy. While the error rate can be a limitation, base-calling methods and bioinformatics analysis can improve the accuracy (Wang et al., 2021). Several studies validated HLA class I typing using ligation-based nanopore sequencing with routine pharmacogenomics diagnostics (Ammar et al., 2015; Ton et al., 2018; Matern et al., 2020; Stockton et al., 2020). However, here, we aimed to compare the effectiveness of two different library preparation methods for nanopore sequencing—transposase-based and ligation-based—with two different primer sets and two data analysis tools for HLA class I typing. We discuss each in terms of accuracy, speed, and cost for routine use in a clinical pharmacogenetics laboratory.

## 2 Materials and methods

### 2.1 Study design and DNA samples

**FIGURE 1:**
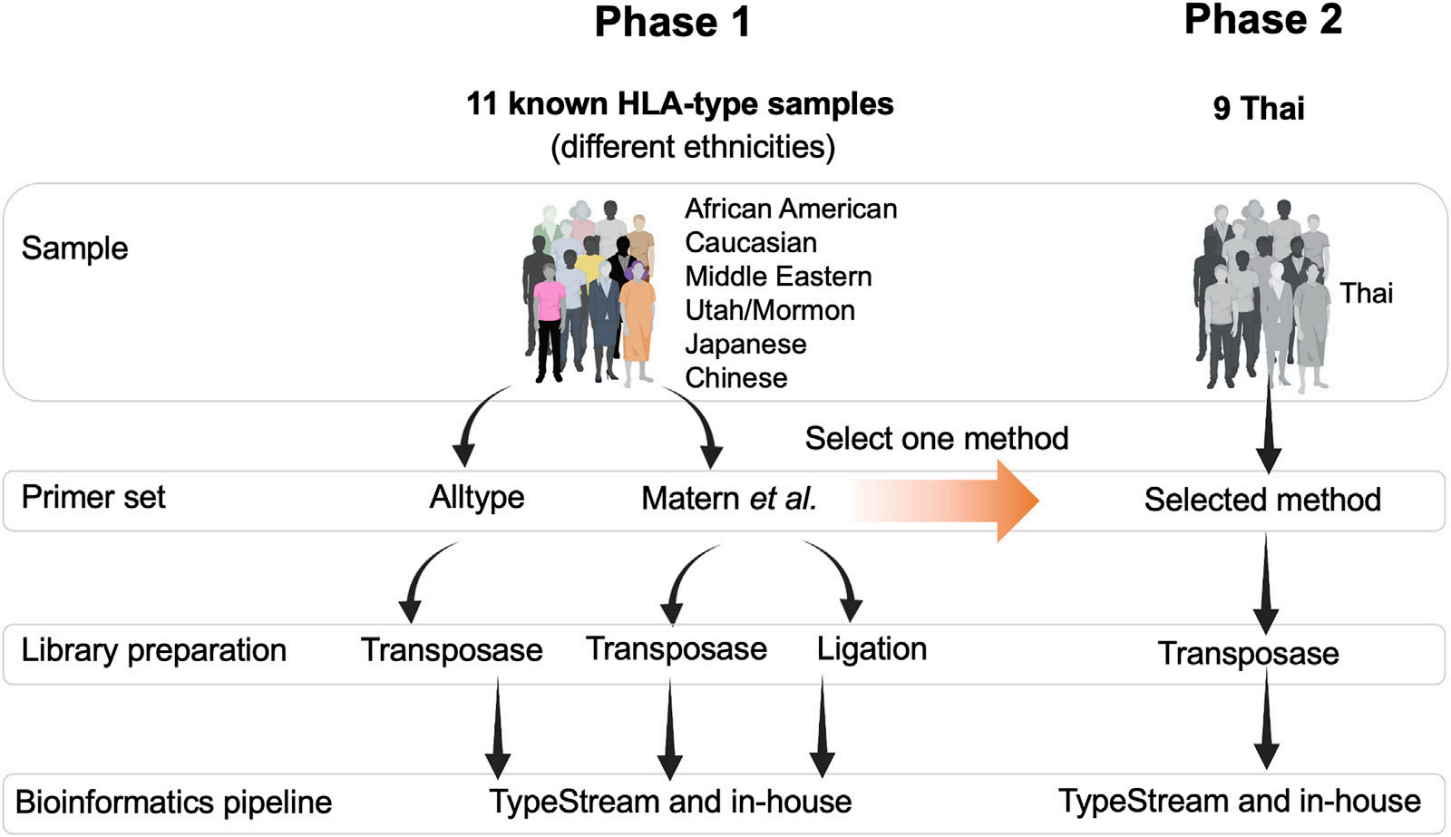
Overall framework of this study. There were two phases of the study: phase 1, testing two sets of primers, two types of DNA library preparation kits, and two bioinformatics pipelines. Phase 2, Thai ethnicity, one set of primer, one type of library preparation kit, and two bioinformatics pipelines.

The study design is shown in Figure 1. Two sets of DNA samples were used in this study: 1) 11 well-characterized DNA standard samples obtained from GeT-RM (Coriell Institute for Medical Research, United States) and 2) nine DNA samples from Thai patients selected from a routine pharmacogenetics laboratory, Faculty of Associated Medical Sciences, Chiang Mai University, and who had previously undergone PCR-SSP. In phase 1, the ALLType primer set was only investigated with transposase because the PCR product obtained from ALLType was not enough to perform both transposase-based and ligation-based methods. Therefore, we only chose transposase-based HLA typing for ALLType. In phase 2, the selected method mentioned in Figure 1 is a primer set providing the best result and is further applied to nine clinical samples. This study was approved by the Research Ethics Committee, Faculty of Associated Medical Sciences, Chiang Mai University, Thailand (approval code: AMSEC-65EX-001; date of approval: 10 January 2022 and AMSEC-65EX-013; date of approval: 8 April 2022).

### 2.2 Polymerase chain reaction sequence-specific primers (PCR-SSP)

This technique was used in the routine pharmacogenetics laboratory at the Faculty of Associated Medical Sciences, Chiang Mai University, to analyze HLA-B*57:01, B*58:01, and B*15:02 markers to predict the risk of drug hypersensitivity. All nine samples (Thai cohorts) were collected from the blood samples remaining from routine laboratory work, without identifiable patient information. DNA was extracted using a NucleoSpin™ Tissue kit (Macherey-Nagel, Germany), following the manufacturer’s instructions. Two hundred microliters of buffy coat were used and eluted in 100 μL of sterile water. The quality of extracted DNA was determined with an Epoch microplate spectrophotometer (BioTek^®^, Winooski, VT, United States) with an absorbance of 260/280. Then, at least 100 ng of DNA was subjected to PCR using primers specific to each marker. All PCR reactions included positive, negative, and non-template controls (NTCs) and internal control primers for the human growth hormone gene (*HGH*) when detecting HLA-B*57:01 and for the interferon-gamma gene (*IFN*) when detecting HLA-B*58:01 and HLA-B*15:02. The primer sequences are shown as follows:

HLA-B*57:*01* (product size: 262 bp and 94 bp) (Cascella et al., 2015).

B57_F GTC​TCA​CAT​CAT​CCA​GGT.

B57_R CGT​CTC​CTT​CCC​GTT​CTC.

B5701_R1 ATC​CTT​GCC​GTC​GTA​GGC​GG.

B5701_R2 ATC​CTT​GCC​GTC​GTA​GGC​AG.

HLA-B*58:*01* (product size: 358 bp) (Virakul et al., 2010).

B58_F ACG​GAA​CAT​GAA​GGC​CTC​C.

B58_R CAG​CCA​TAC​ATC​CTC​TGG​ATG​A.

HLA-B*15:02 (product size: 406 bp and 483 bp) (Suttikham, 2021).

B15_F CTC​CTG​CTG​CTC​TCG​GGA.

B15_R GGC​TCT​CTC​GGT​AAG​TCT​GTG​TGT​T.

B1502_F1 AGC​GAG​TCC​GAG​GAT​GGC.

B1502_R1 GTC​GTA​GGC​GGA​CTG​GTC​ATA.

PCR reactions for all three markers were performed in a total PCR reaction volume of 12.5 μL, containing 100 ng genomic DNA, 6.25 µL Quick Taq^®^ HS DyeMix (Toyobo, Japan), and 0.2 µM each of forward and reverse primers. The PCR conditions are as follows: 1) pre-denaturation at 94°C for 1 min; 2) 30 cycles of denaturation at 94°C for 30 s, annealing at 55°C (HLA-B*57:01), 65°C (HLA-B*58:01), or 60°C (HLA-B*15:02) for 30 s; and 3) extension at 68°C for 30 s to 1 min depending on the product size. The GeneAmp^®^ PCR System 9700 (Applied Biosystems, United States) was used. PCR products were then analyzed with agarose gel electrophoresis (2% agarose).

### 2.3 Long-range PCR and primer sets

HLA-specific primers were obtained from two different sources: 1) as reported by Matern et al., 2020 and 2) purchased commercially from One Lambda ALLType (Thermo Fisher, United States). The first set of primers covers the entire *HLA* gene sequence, from the 5′ UTR region to the 3′ UTR region (expected amplified size: 3,400 bp). The second primer set from ALLType 11-loci covers various sizes of PCR products, ranging from 1 to 8 kb.

Using Matern’s primer set, the 25-μL PCR amplification reaction volume contained 50 ng genomic DNA, 1 μL DNA polymerase (1.25 U per μL) (PrimeSTAR GXL enzyme, Takara Bio, Japan), 5 μL × 5 PrimeSTAR GXL Buffer, 2 μL of 2.5 mM dNTP (200 μM final concentration), 1 μL of 10 μM forward primer, and 1 μL of 10 μM reverse primer (0.2 μM final concentration). The PCR conditions were as follows: primary denaturation at 98°C for 1 min, followed by 30 cycles at 98°C for 10 s, 68°C for 40 s, and a hold at 12°C. Long-range PCR reactions were performed with the Bio-Rad T100 machine (Bio-Rad, United States).

The ALLType assay (One Lambda, United States) began with targeted PCR amplification using commercial primers (no data available), which involved long-range PCRs of the entire *HLA* gene. Starting the DNA input with 100 ng, the PCR primers were added and cycling conditions were set according to the manufacturer’s instructions. The PCR conditions were as follows: 1) one cycle of denaturation at 94°C for 2 min, 2) 22 cycles of 10 s at 98°C and 3 min at 69°C, 3) eight cycles of 10 s at 98°C and 3 min at 60°C, and 4) hold at 4°C. The amplicons were then cleaned using AMPure beads (Beckman, United States). The purified ALLType amplicons were used for ONT transposase-based library preparation. From the PCR amplicons generated from both primer sets, a subset of amplified samples was quantified using a Qubit dsDNA HS kit (Applied Biosystems, United States) and further used for nanopore sequencing.

### 2.4 DNA library preparation and nanopore sequencing

#### 2.4.1 Transposase-based sequencing

The Matern and ALLType amplicons were used as input DNA for the Rapid Barcoding Kit (SQK-RBK004, ONT, UK); amplicons were individually barcoded according to the RBK-004 protocol. The amount of input DNA in each tube was adjusted to 100 ng per sample in 7.5 µL nuclease-free water. Sequencing was performed on a MinION Mk1C nanopore sequencer according to the manufacturer’s instructions. Multiple samples were combined per R9.4.1 flow cell (FLO-MIN106D, ONT, UK).

#### 2.4.2 Ligation-based sequencing

Purified amplicons from Matern were used as input DNA for Native Barcoding (EXP-NBD104 and EXP-NBD114 kits, ONT, UK) combined with the Ligation Sequencing Kit (SQK-LSK109, ONT, UK); amplicons were individually barcoded according to the Native Barcoding Amplicons protocol. The amount of input DNA in each tube was adjusted to 100 ng per sample in 48 µL nuclease-free water.

### 2.5 Data pre-processing and bioinformatics analysis

Nanopore sequencing was performed using MinKNOW software version 22.05.8, which collected read data in the form of FAST5 files. The electrical signal data from FAST5 files were then converted into nucleotide reads and demultiplexed using Guppy version 5.0.16 (ONT) with a super accuracy base-calling model. Next, the reads were trimmed for adapter and barcode sequences and filtered for a length greater than 1,000 bp using Porechop version 0.2.4 (Wick et al., 2017). Minimap version 2.2.4 (Li, 2021) was used to map the reads to HLA reference sequences for *HLA-A*, *HLA-B*, and *HLA-C* genes, which were downloaded from the IPD-IMGT/HLA database version 3.48 (https://www.ebi.ac.uk/ipd/imgt/hla/). We also included all pseudogenes (i.e., HLA-Y, HLA-E, and HLA-F) in the reference sequences to remove off-target reads. Only primary aligned reads that soft-clipped bases less than 150 bp were kept. This step helped remove off-target reads that may have been present in the dataset. Finally, reads that passed the cut-off were used for downstream analysis.

### 2.6 Data analysis and HLA typing

#### 2.6.1 TypeStream

The passed reads were aligned to the HLA reference sequences and analyzed with TypeStream Visual NGS (next-generation sequencing) software (version 2.1.0.40; One Lambda) and the IPD-IMGT/HLA database (https://www.ebi.ac.uk/ipd/imgt/hla/).

#### 2.6.2 In-house data analysis

The passed reads were corrected for random errors with Canu version 2.2 (Koren et al., 2017). Corrected reads were then mapped to the HLA reference sequences, and variants were called using PEPPER–Margin–DeepVariant version r0.8 (Shafin et al., 2021). All variants were phased using WhatsHap version 1.5 (Patterson et al., 2015), resulting in phased variant-call format (VCF) and phased binary-alignment (BAM) files. The reads in each haplotype block were extracted from phased BAM files and polished using Racon version 1.5.0 (Vaser et al., 2017), followed by Medaka version 1.7.0 (Medaka, 2018), respectively. The consensus sequences obtained from Medaka were aligned with the IPD-IMGT/HLA database using BLAST version 2.13 (Altschul et al., 1990). The best matches with the highest bit-score and no mismatches were selected for the final assignment of each HLA allele.

## 3 Results

### 3.1 HLA amplification from DNA standards using two different primer sets

**FIGURE 2:**
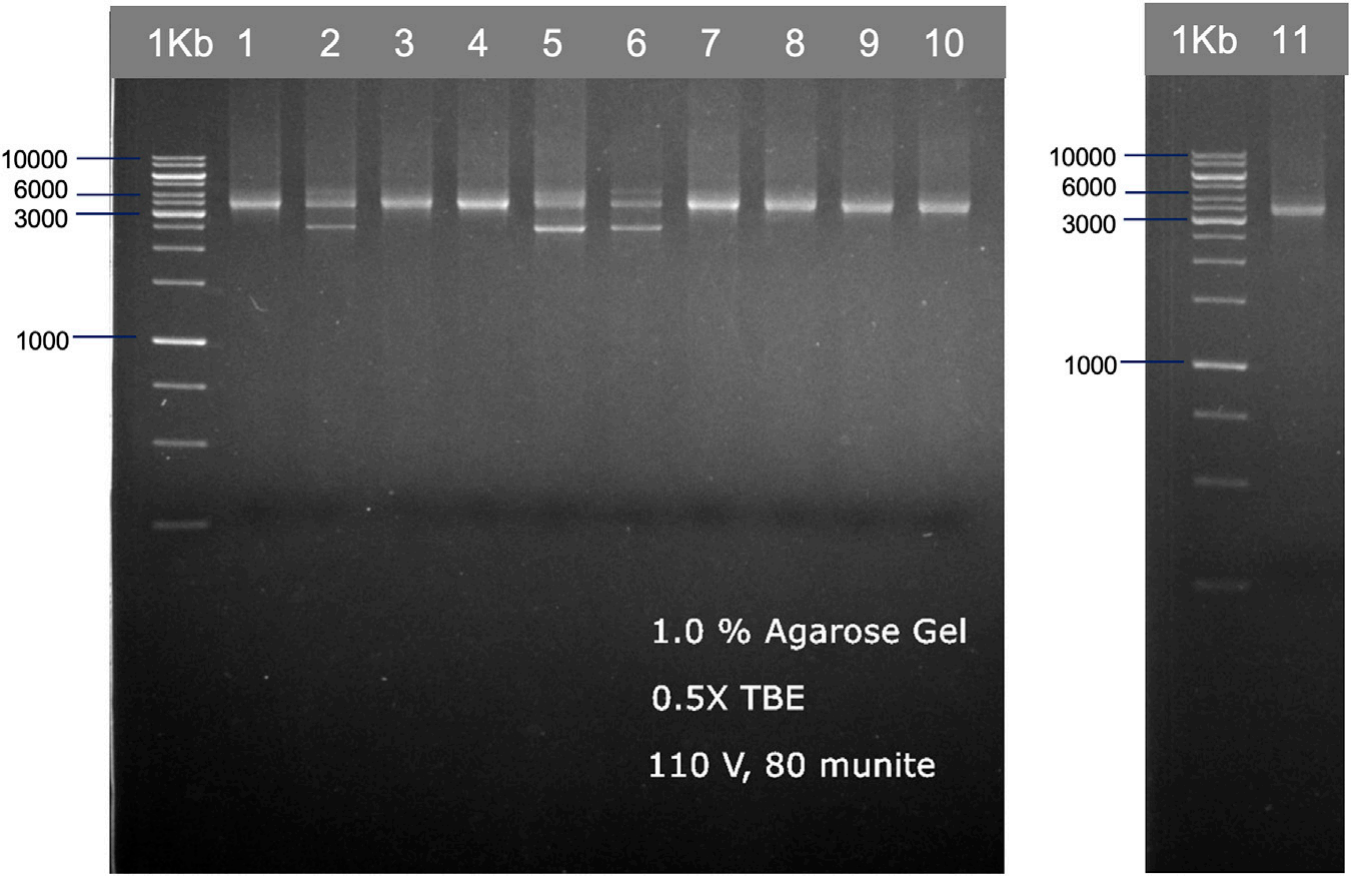
Examples of HLA-A, HLA-B, and HLA-C PCR products from 11 individuals, which are shown in two gel electrophoresis images. The PCR products were ran on 1.0% agarose gel electrophoresis, generated from the published primer set (Matern et al., 2020) with our modified PCR protocol.

We used two different primer sets—by a set from ALLType and a set from the previously published study by Matern et al., 2020, to amplify the HLA loci from 11 DNA standards. For the first primer set, ALLType, the manufacturer’s protocol was followed for long-range PCR, which takes around 3 h. PCR products from the ALLType primer set were quantified with the PCR product using Qubit, and the concentration varied from 5 to 25 ng/μL. For primers published by Matern et al., we did not follow the original protocol as published, instead different PCR conditions and polymerase enzymes were used to reduce the cost of PCR amplification and develop a simple protocol. Only one PCR reaction was performed by combining three sets of primers; the expected band size for HLA-A, -B, and -C was 3.4 kb (DNA amplicon approximately 80–150 ng). However, some samples showed an additional band at 2.5 kb (Figure 2). All amplicon bands were then used for transposase-based and ligation-based nanopore sequencing.

### 3.2 HLA class I typing of DNA standard samples

**FIGURE 3:**
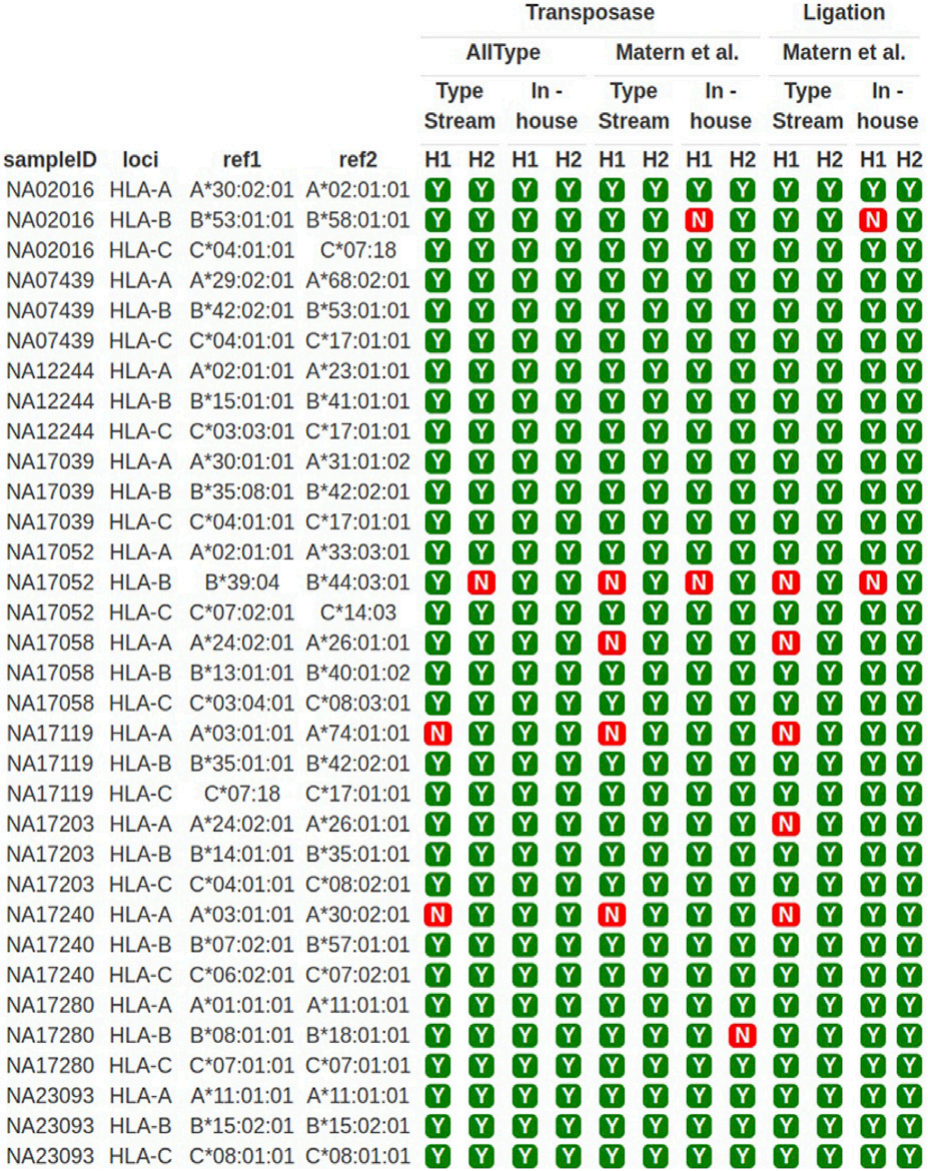
*HLA-A*, *-B*, and *-C* typing of 11 well-characterized samples. Y means concordant *HLA* haplotype compared to GeT-RM standard; N means discordant *HLA* alleles compared to GeT-RM standard; H1 means haplotype 1; H2 means haplotype 2 (more details are shown in Supplementary Table S1).

The sequencing results obtained from both primer sets were used for HLA typing analysis. TypeStream commercial software and our in-house bioinformatics pipeline were applied. The result of HLA class I typing is shown in Figure 3. Highlighted data with “Y” (yes) show 3-field allele concordance to the HLA haplotype reported for the GeT-RM standard, and “N” shows discordant data observed. The success rate of HLA typing from each pipeline varied. The ALLType primer set combined with transposase-based sequencing gave 100% accuracy based on comparison to the GeT-RM standard as analyzed with our in-house bioinformatics tool, while 91% accuracy was obtained from the TypeStream bioinformatics tool. In contrast, discrepancies were revealed with the Matern et al. primer set combined with transposase-based sequencing, where the sequencing accuracy was 87%, analyzed with TypeStream, and 91%, analyzed with our in-house analysis tool. Using ligation-based sequencing, the accuracy was 85%, analyzed with TypeStream, and 94%, analyzed with our in-house bioinformatics tools, which are not significantly different from transposase-based sequencing. *HLA-A* and *HLA-B* analysis exhibited more faults than *HLA-C* analysis. Our in-house bioinformatics pipeline worked well with both library preparation kits and worked best with the ALLType primer set. The efficiency of transposase-based sequencing was comparable to that of ligation-based sequencing but reduced the hands-on time. Overall, the ALLType primer set with transposase-based nanopore sequencing provided the most rapid approach and accurate results comparable to ligation-based results; this approach was then applied to the clinical samples.

### 3.3 HLA class I typing of clinical samples

We then performed HLA typing on nine clinical samples. We used the ALLType primer set and performed transposase-based nanopore sequencing, followed by analysis with TypeStream and our in-house bioinformatics pipeline. PCR-SSP had been performed on these samples previously in a routine pharmacogenomics laboratory. Highlighted data show discrepancies between bioinformatics pipelines (TypeStream and in-house) (Table 1). Eight out of nine samples produced the same results, while the remaining one sample revealed a discrepancy in the *HLA-C* locus. Sample PAT2 showed only one *HLA-C* allele mismatch. For sample PAT4, haplotype 1 could be HLA-B*07:06:01 or HLA-B*07:05:01.

**TABLE 1:** HLA-A, -B, and -C typing of nine Thai patient samples.

Sample	CMU_ID	Locus	HLA-B*15:02/58:01/57:01 (PCR-SSP)	Transposase (ALLType)			
				TypeStream		In-house	
				Haplotype 1	Haplotype 2	Haplotype 1	Haplotype 2
		HLA-A	NT	A*11:01:01	A*11:01:01	A*11:01:01	A*11:01:01
PAT1	CMU2	HLA-B	neg	B*51:01:01	B*46:01:01	B*51:01:01	B*46:01:01
		HLA-C	NT	C*14:02:01	C*01:02:01	C*14:02:01	C*01:02:01
		HLA-A	NT	A*02:01:01	A*33:03:01	A*02:01:01	A*33:03:01
PAT2	CMU3	HLA-B	B*58:01	B*51:01:01	B*58:01:01	B*51:01:01	B*58:01:01
		HLA-C	NT	C*01:211N	C*03:02:02	C*01:02:01	C*03:02:02
		HLA-A	NT	A*68:01:02	A*33:03:01	A*68:01:02	A*33:03:01
PAT3	CMU4	HLA-B	B*58:01	B*58:01:01	B*52:01:01	B*58:01:01	B*52:01:01
		HLA-C	NT	C*07:02:01	C*03:02:02	C*07:02:01	C*03:02:02
		HLA-A	NT	A*02:03:01	A*11:01:01	A*02:03:01	A*11:01:01
PAT4	CMU8	HLA-B	B*15:02	B*07:06:01/07:05:01	B*15:02:01	B*07:06:01	B*15:02:01
		HLA-C	NT	C*08:01:01	C*07:02:01	C*08:01:01	C*07:02:01
		HLA-A	NT	A*11:01:01	A*11:01:01	A*11:01:01	A*11:01:01
PAT5	CMU9	HLA-B	neg	B*15:450	B*40:01:02	B*15:450	B*40:01:02
		HLA-C	NT	C*03:04:01	C*12:02:02	C*03:04:01	C*12:02:02
		HLA-A	NT	A*74:02:01	A*11:01:01	A*74:02:01	A*11:01:01
PAT6	CMU10	HLA-B	neg	B*48:01:01	B*15:21:01	B*48:01:01	B*15:21:01
		HLA-C	NT	C*08:22:01	C*04:03:01	C*08:22:01	C*04:03:01
		HLA-A	NT	A*11:01:01	A*11:01:01	A*11:01:01	A*11:01:01
PAT7	CMU12	HLA-B	neg	B*40:01:02	B*18:02:01	B*40:01:02	B*18:02:01
		HLA-C	NT	C*07:04:01	C*07:02:01	C*07:04:01	C*07:02:01
		HLA-A	NT	A*11:01:01	A*02:03:01	A*11:01:01	A*02:03:01
PAT8	CMU13	HLA-B	B*15:02	B*15:02:01	B*13:01:01	B*15:02:01	B*13:01:01
		HLA-C	NT	C*08:01:01	C*04:03:01	C*08:01:01	C*04:03:01
		HLA-A	NT	A*11:01:01	A*02:07:01	A*11:01:01	A*02:07:01
PAT9	CMU14	HLA-B	B*15:02	B*46:01:01	B*15:02:01	B*46:01:01	B*15:02:01
		HLA-C	NT	C*01:02:01	C*08:01:01	C*01:02:01	C*08:01:01

NT, not tested; neg, no HLA-B*15:02/58:01/57:01; gray highlights indicate discordance results obtained from TypeStream and in-house bioinformatics tools. The HLA genotype had been shown in italic.

### 3.4 Turnaround time and cost of each pipeline

**FIGURE 4:**
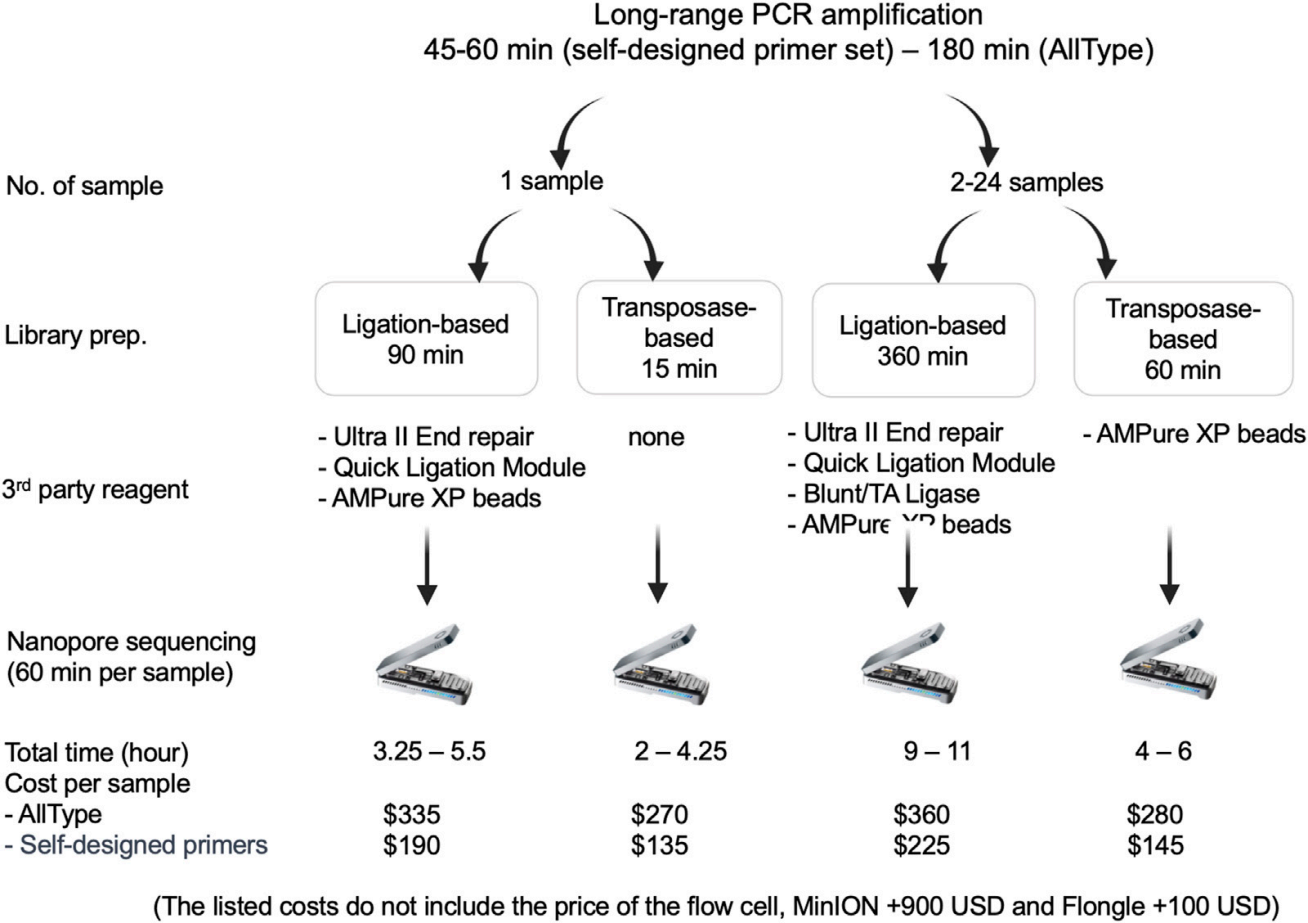
Materials used and time required for ligation-based and transposase-based HLA typing.

The speed and cost of ligation-based nanopore sequencing was compared to that of transposase-based nanopore sequencing using the ALLType primer set (Figure 4). With the transposase-based sequencing approach and ultrarapid barcoding, the overall hands-on time for one sample decreased from 5.5 h to 4.25 h, compared to ligation-based nanopore sequencing. For more than two samples, transposase-based sequencing can be completed in 4–6 h, which is much quicker than the ligation-based approach. For the cost of each assay, the transposase**-**based approach uses less third-party reagents than the ligation**-**based approach listed in Figure 4. Therefore, the cost per sample of the transposase-based approach was approximately 270 USD, which is less than the 335 USD required for the ligation-based approach. It should be noted that both approaches need an additional 900 USD for the MinION flow cell or an extra 100 USD for the Flongle flow cell**.**

## 4 Discussion

There is significant genetic variation in human *HLA* genes across ethnic populations, affecting the selection of predictors for drug hypersensitivity reactions. HLA-associated pharmacogenomic markers have become more common for being genotyped before treatment with specific drugs. The Clinical Pharmacogenetics Implementation Consortium (CPIC) guideline (https://cpicpgx.org/guidelines/) provides recommendations for the use of drugs that often cause severe allergic reactions (SCARs) in the context of *HLA* genotypes, describing both how to interpret the genotype results and how physicians should select alternative therapies. The Pharmacogenomics Knowledgebase (PharmGKB) (http://www.pharmgkb.org) provides information about the impact of genetic variation on drug response. For some drugs, the recommendation is to genotype more than one *HLA* allele; for example, HLA-B*15:02 and/or HLA-A*31:01 should be tested for preventing carbamazepine- and oxcarbazepine-induced SCARs, as both the alleles are normally found in different frequencies according to the geographical distribution (Phillips et al., 2018). In Thailand, the national policy is to test for HLA-B*15:02, as it is mostly found in Southeast Asian populations; some less prevalent alleles, including HLA-B*15:08, HLA-B*15:11, and HLA-B*15:21, are not yet included in routine laboratory testing using specific primer sets. Considering the limitation of the cost per test and the availability of allele-specific test kits in the market, only HLA-B*15:02 is selected for all patients, but this might cause false-negative results in the context of SCARs. However, some laboratories in Thailand may provide high-resolution *HLA-B* typing and report the possibility of *HLA-B*-induced SCARs.

In Thailand, PCRs and other techniques are predominantly used for HLA genotyping in pharmacogenomics laboratories due to their lower cost and less complex data analysis. However, these methods often lack the resolution and accuracy that DNA sequencing technologies can provide, which is one of the reasons we are exploring nanopore sequencing in our study. Sequencing technologies indeed have the potential to greatly change the paradigm of pharmacogenomics such as 1) resolution and accuracy, 2) scale and throughput, and 3) discovery of novel variants.

Here, we investigated the use of transposase-based nanopore sequencing for HLA class I typing, focusing on its real-world application in pharmacogenomics laboratories. DNA standard samples from people of different ethnicities and clinical samples from Thai patients were tested to determine the effectiveness of our modified method, without ethnicity bias. Using the ALLType primer set with TypeStream or our in-house bioinformatics tools, we achieved more than 90% accuracy in genotyping, specifically for HLA-B*15:02, HLA-B*58:01, HLA-B*57:01, and HLA-B*53:01 in standard samples and provided 3-field alleles at high resolution. With the overall hands-on time, comparing transposase- and ligation-based processes, the transposase-based method can reduce the hands-on time from 5.5 h to 4.25 h in one sample while running. Our results were similar to those of a previous study of SARS-CoV-2 RNA sequencing, in which the sequencing process time was reduced by native barcoding (ligation-based), which approximately takes 9 h–7 h by using rapid barcoding (transposase-based) (Constantinides et al., 2021). We also conducted tests employing PCR products from two samples without bead cleaning. Remarkably, we observed favorable sequencing results and an adequate number of reads for HLA typing. This success can be attributed to the utilization of a mere 2 µL of the PCR product, which was mixed with 5.5 µL of nuclease-free water. Consequently, the dilution effectively mitigated the presence of any potential sequencing inhibitors, enabling us to obtain the desired sequencing reads without bead cleaning. For the cost per sample, use of the ALLType primer set cost 270 USD per sample (plus 900 USD MinION flow cell or 100 USD Flongle flow cell) for transposase-based sequencing *vs.* 335 USD per sample for ligation-based sequencing. Moreover, to lower the cost per sample, the published primer set and our modified PCR protocols were tested in place of the commercial ALLType primer set. Even without 100% success, up to 94% accuracy was achieved. In countries with low-to-middle income levels, like Thailand, HLA-class I sequencing using in-house primers is currently priced at 266 USD. However, employing in-house primers with a transposase-based nanopore sequencing approach reduces the cost to 235 USD (included Flongle flow cell cost) without the need to wait for multiple samples to be combined; this also provides a faster turnaround time. It is worth noting that the R9.4.1 flow cell and the RBK-004 kit were used in this study. Both will be replaced by new versions of the flow cell (i.e., R10 version HD) and Kit 14 chemistry, which are designed as they deliver higher consensus accuracy.

When the clinical samples were analyzed using transposase-based sequencing, we obtained only one discordant in the second-field result of *HLA-C* (PAT2) when comparing TypeStream and our in-house bioinformatics pipeline. Moreover, in sample PAT4, the HLA-B*15:02 allele was detected using two different bioinformatics tools, but another allele was identified as either B*07:06:01 or *07:05:01* with TypeStream, but only one allele B*07:06:01 was assigned with our in-house analysis tool. When sample PAT4 was then assayed with the PCR-SSOP test kit (LABType, One Lambda, Thermo Fisher, United States) (data not shown), HLA-B*07:05:01:01 was detected, with several other possible alleles reported (e.g., *07:05/07:06/07:90/07:105*). In the Allele Frequency Net Database (http://www.allelefrequencies.net/), these alleles, including B*07:06, are suggested to be rare alleles in Thai, Taiwanese, and Chinese Northern Han populations, while HLA-B*07:05 is found to be in 1.4%–2% of the Thai population. Therefore, when reporting the most plausible allele from PCR-SSOP HLA-B typing in this case, HLA-B*07:05 would be selected rather than other rare alleles because the sample was from a Thai patient.

Interestingly, the PAT6 sample was genotyped as HLA-B*15:02-negative with routine PCR-SSP; therefore, the patient was reported to have a normal risk of carbamazepine-induced Stevens–Johnson syndrome/toxic epidermal necrolysis (SJS/TEN) according to the CPIC guidelines, and carbamazepine would be prescribed as per standard dosing guidelines. However, sequencing-based HLA-B typing identified this patient to have HLA-B*15:21, which is a member of the HLA-B75 serotype and shares structural similarity and peptide-binding specificities with HLA-B*15:02, reported to be associated with carbamazepine-induced SJS/TEN (Jaruthamsophon et al., 2017). Therefore, if laboratories provided HLA-B typing with high-resolution results, instead of testing only for specific alleles, alternative drugs could be considered for this patient to reduce the risk of a severe hypersensitivity reaction. This is a good example of the importance of selecting appropriate methods in a routine laboratory setting.

Another highlight of long-read sequencing is the high resolution of the HLA typing result. The data from two primer sets and of both library preparation methods were reported through HLA alleles in 3- or 4-field compared to the conventional PCR method, which provides normally in 1- or 2-field. The accuracy and the level of fields of HLA typing in pharmacogenomics are crucial. With current known allele associations, at least 2-field is required to identify the *HLA* gene group; however, if the 3- or 4-field is known, the benefits will be in identifying the specific *HLA* allele within the group and gaining more precise data to help identify and conclude rare or novel alleles associated with particular drug hypersensitivity reactions. Even high resolution of the HLA typing result with 3- or 4-field can be retrieved from several next-generation sequencing platforms including short-read sequencing (Illumina) or long-read sequencing (PacBio and ONT), with ambiguity reduction compared to Sanger sequencing; NGS-based HLA typing may type *HLA* alleles on each chromosome at higher resolutions, but it is also constrained by read length and read depth because the HLA system is so highly polymorphic (Bauer et al., 2018). ONT can enhance the *de novo* assembly, mapping accuracy, and structural variation detection (Amarasinghe et al., 2020) and has been released via various library preparation methods that depend on studies and flexible to be applied for reducing the hands-on time and be cost-effective in routine diagnostics. On the other hand, high-resolution HLA typing data from long-read sequencing were applied to the organ or stem-cell transplantation and blood transfusion. Moreover, the data can predict the genetic association with other diseases.

## 5 Conclusion

Our study suggests that transposase-based nanopore sequencing could potentially be a prospective screening test in pharmacogenomics laboratories. Compared to other sequencing technologies, the significant cost difference due to hardware is a prominent feature that makes nanopore sequencing more accessible, especially for smaller laboratories or those in developing countries. It constitutes an ethnicity-independent, rapid, and cost-effective method for identifying significant-risk alleles reducing the risk of severe, potentially fatal SCARs and reducing the turnaround time and cost per sample compared to other sequencing techniques.

## Data Availability

The original contributions presented in the study are publicly available. These data can be found here: PRJEB61855.
