# Supplementary material for: Ultrarapid and high-resolution HLA class I typing using transposase-based nanopore sequencing applied in pharmacogenetic testing
Source: Front Genet. 2023 Jun 23;14:1213457. doi: 10.3389/fgene.2023.1213457 (PMC10326273; doi:10.3389/fgene.2023.1213457)
Supplement: Supplementary file 1 [file DataSheet1.PDF]

Transposase-based HLA-A, HLA-B, HLA-C Sequencing Using Oxford Nanopore Technologies	Protocol
	Thidathip **Wongsurawat**<br>Thidathip.won@mahidol.edu
	Date: 20/05/2023

1 Purpose

1.1 To sequence HLA A, B, C using MinION flow cell

2 Objective

2.1 To pool and check PCR amplicons obtaining from HLA-A, HLA-B, HLA-C amplification (in case user perform PCR amplification of each locus in a separate tube)

2.2 To perform transposase-based sequencing using Rapid Barcoding Kit by Oxford Nanopore Technologies (ONT).

3 Materials and Consumables

3.1 PCR amplicons

3.2 Rapid Barcoding Kit: RBK004 or RBK114.24

3.3 MinION flow cell R9.4.1 or R10.4.1

3.4 Nuclease-free water (B1500L, NEB)

3.5 100% Ethanol (prepare from 200 proof Ethanol 100%, RNase-free; Fisher Cat. BP2818-500)

3.6 Freshly-prepared 80% ethanol in nuclease-free water (3 ml total)

3.7 Qubit™ dsDNA BR Assay Kit (Catalog number: Q32850)

3.8 1.5 mL microfuge tube (Eppendorf™ 022431021) and rack

3.9 Qubit™ Assay Tubes (Q32856) (in case user needs to check the concentration of the PCR amplicon concentration)

3.10 5 ml Eppendorf DNA LoBind tubes

3.11 PCR strip (Thermo Scientific™ ABgene™ EasyStrip™ AB2000)

3.12 Filter tip 10 μl, 20 μl, 200 μl, 1000 μl

3.13 Pipette set (1000, 200, 100, 10 μl)

3.14 Magnetic bead (Beckman Coulter: A63881) – store at 4°C (DO NOT FREEZE)

3.15 Biohazard bag

3.16 Gloves

3.17 Microfuge

3.18 Vortex mixer

3.19 Thermal cycler (PCR machine)

3.20 Qubit™ Fluorometer

3.21 Ice bucket with ice

3.22 Spin down centrifuge (for PCR strip and 1.5 ml tube)

3.23 Magnetic separation rack (0.2 μl and 1.5 ml) (NEB: S1506S)

3.24 Timer

3.25 MinION MK1B/ Mk1C/ GridION/ Flongle adaptor (for Flongle flow cell)

3.26 Computer PC/ Laptop (8 or 16 Gb RAM, 500 Gb space available)

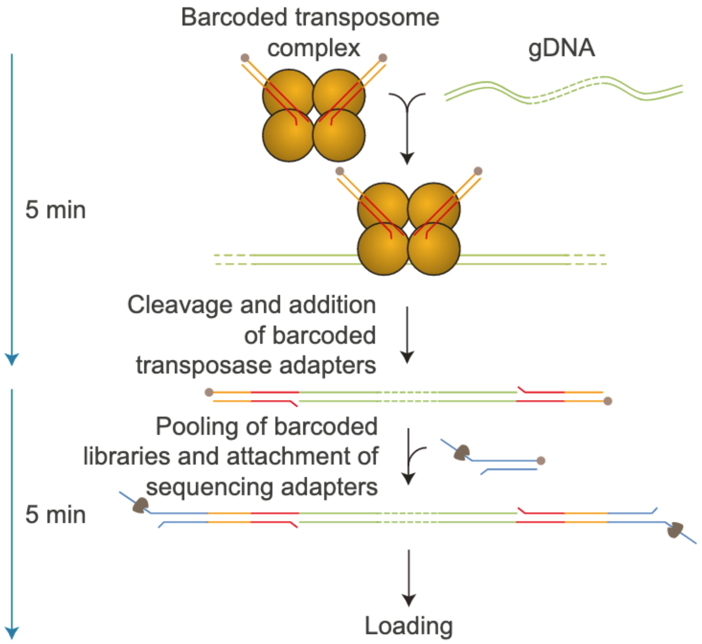

Library preparation: Rapid Barcoding Kit
Ref: https://community.nanoporetech.com/docs/prepare/library_prep_protocols/

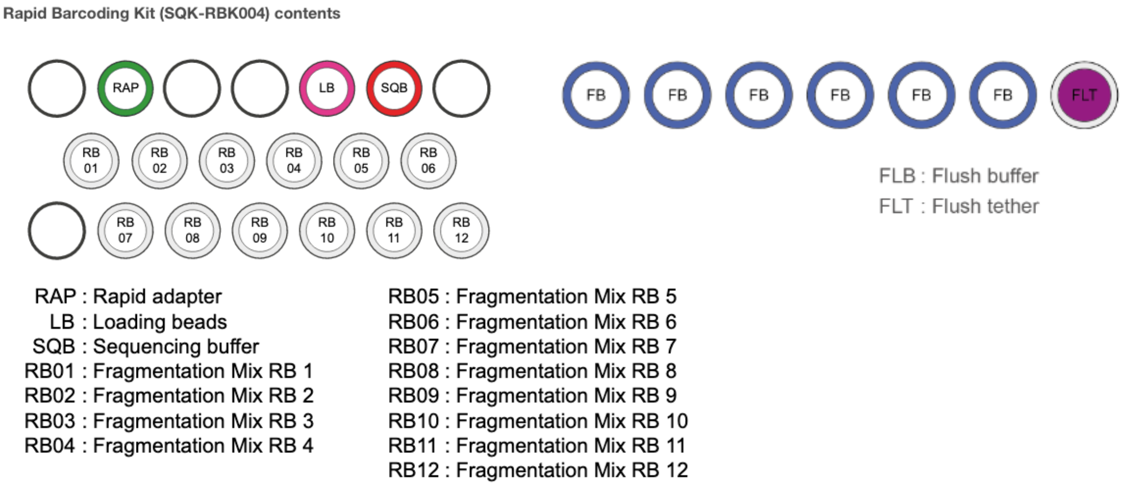

Rapid Barcoding Kit (SQK-RBK004) contents

Thidathip **Wongsurawat**
Thidathip.won@mahidol.edu

4 Procedure

PCR amplification of HLA-A, HLA-B, HLA-C can be performed using AllType™ protocol (https://www.thermofisher.com/onelambda/wo/en/products.html?articleNumber=ALL-11L) or Matern et al.'s protocol (see reference). DNA concentration of PCR amplicon should be at least 20 ng/µl by Qubit assay** (see below) PCR cleanup could be performed before DNA library preparation (optional).

4.1 Pool amplicons

Pool HLA-A, HLA-B, HLA-C amplicon into one tube

☐ Spin down PCR tube

☐ Take 3 µl HLA-A, 3 µl HLA-B, 3 µl HLA-C amplicon and mix by pipette

☐ In a new tube, prepare 5.5 µl NFW. Then add 2 µl pooled amplicon in to NFW

Mix by pipetting and spin down

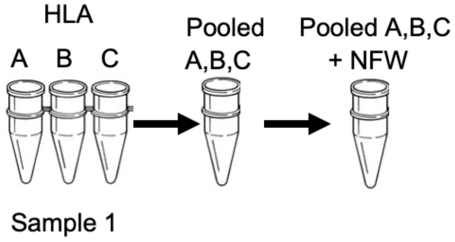

4.2 Transposase cutting (RBK004)

☐ Set up PCR at 30°C: 1min and 80°C: 1 min

☐ Add 2.5 µl Rapid Barcode RB01-12 (one for each sample, included in kit). Mix gently by flicking the tube, and spin down

☐ Incubate at 30°C: 1min and 80°C: 1 min in thermocycler

☐ If >3 samples, **Ampure bead cleanup***** (see below) 1:1 (120:120) ratio, wash by freshly-prepared 80% Ethanol and resuspend pellet in 15 µl NFW and flicking. Then incubate for 2 min at room temp. If 1-3 samples, combine all sample (4 µl each) in to a new tube please move to the next step.

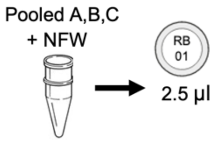

Temp.	Time
30°C	1 min
80°C	1 min
20°C	hold

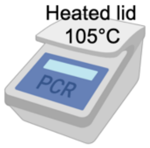

4.3 Adaptor attachment

☐ Add 1 µl RAP into pooled barcoded DNA. Mix gently by flicking the tube. Incubate 5 mins.

☐ Thaw the SBII, LBII, FLT and FB at room temperature

4.4 Priming and loading flow cell

☐ Vortex SBII, LBII, FLT and FB and spin down

☐ In a new tube, prepare the library for loading as follows:

35 µl SBII

25.5 µl LBII,

15 µl DNA library

☐ Prepare priming mix, add 30 µl of FLT directly to the tube of FB, and mix by vortexing.

☐ Set a P1000 pipette to 200 µl & open priming port

☐ Turn the wheel until you can see a small volume of yellow buffer entering the pipette tip (~20-30 µl)

☐ Load 800 µl of the priming mix into the flow cell via the priming port, avoiding the introduction of air bubbles. Wait for 5 minutes.

☐ Lift the SpotON sample port cover & load 200 µl of the priming mix into the flow cell via the priming port (not the SpotON sample port!!!!)

☐ Add 70 µl of sample to SpotON port

☐ Close the SpotON port & priming port → Start sequencing (select RBK004 for the kit)

4.5 In-house data analysis

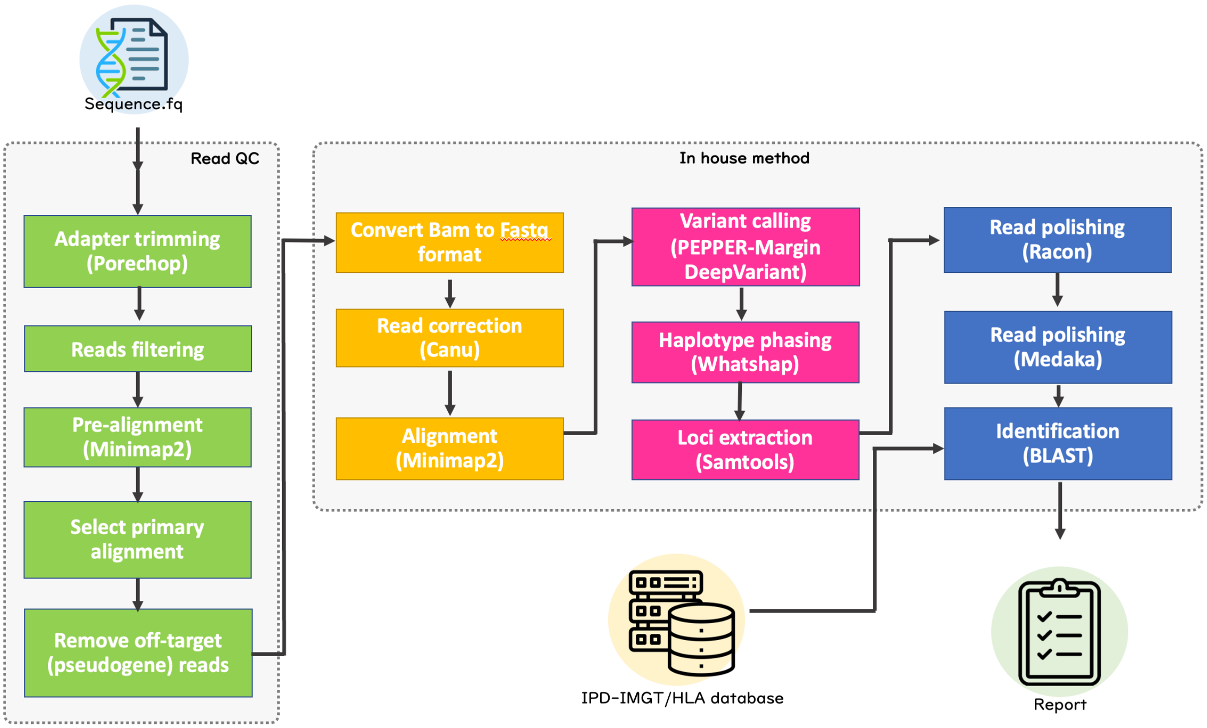

In-house data analysis workflow

☐ Correct passed reads with Canu (Koren et al., 2017).

☐ Map corrected reads to the HLA reference sequences, and variants call by PEPPER-Margin-DeepVariant (Shafin et al., 2021).

☐ Phase variants with Whatshap version 1.5 (Patterson et al., 2015), resulting in phased variant call format (VCF) and phased binary alignment (BAM) files.

☐ Extract reads in each haplotype block from the phased BAM files and polish with Racon version (Vaser et al., 2017), followed by Medaka version (Medaka, 2018), respectively.

☐ Align the consensus sequences obtained from Medaka with the IPD-IMGT/HLA database using BLAST (Altschul et al., 1990).

☐ Select the best matches with the highest bit-score and no mismatches for the final assignment of each HLA allele.

** **Determining DNA concentration using Qubit**

- *Concentration:* Apply 1 µl of DNA. Use the Qubit™ to measure concentration (see ref. 8)
- *Integrity:* Apply 1-5 µl of DNA. Use the 0.8% Agarose gel to check the integrity (reference no. 4). Load 1 kb DNA ladder on the first lane and high molecular weight DNA ladder on the last lane. After running the gel electrophoresis, high integrity of DNA will locate above 10 kb in the area of high molecular weight DNA ladder.

*** **Bead clean up using Ampure beads** (modified from reference no. 2)

- Allow magnetic bead reagent to come to room temperature prior to use.
- Mix the reagent well before use. It should appear homogenous and consistent in color (called mixture).
- Add 1 volume of the mixture to 1 volume of sample. For example, adding 80 µl bead to 80 µl DNA amplicons.
- Gently pipette up and down 10 times and gently flick the tube for 5 times then spin down (1 second).
- Incubate the mixture at room temperature for 5-10 minutes.
- Place the mixture onto magnetic separation rack for 3 minutes to separate beads from solution
- Prepare fresh 80% ethanol (800 µl ethanol and 200 µl NFW).
- Aspirate the cleared solution from the reaction tube and discard. This step must be performed while the reaction tube is situated on the magnetic separation rack. Do not disturb the area of magnetic beads.
- Dispense 200 µl of 80% ethanol to reaction tube and incubate for 1 minute at room temperature. Aspirate out the ethanol and discard. Repeat for a total of two washes.
- Remove the tube from magnetic separation rack and spin down the tube (1 second). Then, place the tube onto magnetic separation rack and use 10 µl pipette to aspirate out the remained ethanol and discard.
- Place the reaction tube on bench top to air-dry for 1 minute. Be sure to allow the dry
- Add 15 µl of nuclease free water to the tube of the reaction and flicking 5 times or until no clump and perform spin down (1-2 second). Incubate at room temperature for 2 minutes.

- Place the tube onto magnetic separation rack and use 10 µl pipette to aspirate out the DNA in the NFW and put into the new tube (with label the tube).

5 Safety

5.1 Waste solution must be stored in a biohazard waste container / bag.

5.2 Be careful to minimize generation of aerosol by pipetting.

References

1. Matern, B. M., Olieslagers, T. I., Groeneweg, M., Duygu, B., Wieten, L., Tilanus, M. G. J., and Voorter, C. E. M. (2020). Long-read nanopore sequencing validated for human leukocyte antigen class I typing in routine diagnostics. *J Mol Diagn*. 22, 912-919. doi: 10.1016/j.jmoldx.2020.04.001
2. Koren, S., Walenz, B. P., Berlin, K., Miller, J. R., Bergman, N. H., and Phillippy, A. M. (2017). Canu: scalable and accurate long-read assembly via adaptive k-mer weighting and repeat separation. *Genome Res*. 27 (5), 722-736. doi: 10.1101/gr.215087.116
3. Shafin, K., Pesout, T., Chang, P.-C., Nattestad, M., Kolesnikov, A., Goel, S., et al. (2021). Haplotype-aware variant calling with PEPPER-Margin-Deep Variant enables high accuracy in nanopore long-reads. *Nature Methods*. 18 (11), 1322-1332.
4. Patterson, M., Marschall, T., Pisanti, N., van Iersel, L., Stougie, L., Klau, G. W., and Schönhuth, A. (2015). WhatsHap: weighted haplotype assembly for future-generation sequencing reads. *J Comput Biol*. 22 (6), 498-509. doi:10.1089/cmb.2014.0157
5. Altschul, S. F., Gish, W., Miller, W., Myers, E. W., and Lipman, D. J. (1990). Basic local alignment search tool. *J Mol Biol.* 215 (3), 403-410. doi:10.1016/s0022-2836(05)80360-2
6. Medaka, O. (2018). Sequence correction provided by ONT Research. GitHub https://github. com/nanoporetech/medaka.
7. Vaser, R., Sović, I., Nagarajan, N., and Šikić, M. (2017). Fast and accurate de novo genome assembly from long uncorrected reads. *Genome Res*. 27 (5), 737-746.
8. https://genome.med.harvard.edu/documents/sequencing/Agencourt_AMPure_Protocol.pdf
9. http://tools.thermofisher.com/content/sfs/manuals/qubit_assays_qrc.pdf
